# Letter from Dr. Hall

**Published:** 1849

**Authors:** 


### ARTICLE I.

#### LETTER FROM DR. HALL.

The following letter from our much esteemed correspondent, Dr. Hall, solves the difficulty which we had, at the time of publication, in accounting for the symptoms in the case reported by him in No. 4, of this volume of our journal.

We find upon again comparing his manuscript, with the report of the case, as published, that the mistake referred to was *not* made by the compositor. H.

TOULON, Illinois, February 10, 1849.

GENTLEMEN :—There is a material error in the description of the case of cerebal abscess, published in your journal; and whether the error is mine, or the compositor's, I know not; my notes are correct, from which I transcribed it. *The Paralysis was on the left side*—in strict accordance with the general rule in cerebral disease.

I was surprised, in reading the letter of your Paris correspondent, to find some observations on Jobert's *new method* of treating fractures without splints and bandages; for there is nothing particularly new about it. In 1834 Mr. Wardrop, in a clinical lecture, made some observations on the injurious effects of splints and bandages, and the management of fractures without them ; this lecture was published in the London Lancet, Article 4th, 1834 ; Page 55. It made no impression, or next to none. In the Lancet for October 21, 1835, page 168, there is an essay on the same subject, by Mr. Radley, of of Newton Abbot, Devonshire. It was followed up by some three or four more from the same pen, with cases illustrative the advantages the new method possessed. These essays were plainly and forcibly written, and consequently produced an impression; and the principles of the treatment were debated in the London Medical Society, on November 30, 1835, and a few following meetings and published in the Lancet.

THOMAS HALL.

